# Supplementary material for: Population-level prevalence of detectable HIV viremia in people who inject drugs (PWID) in Ukraine: Implications for HIV treatment and case finding interventions
Source: PLoS One. 2023 Oct 26;18(10):e0290661. doi: 10.1371/journal.pone.0290661 (PMC10602286; doi:10.1371/journal.pone.0290661)
Supplement: Attachment — Submitted filename: PONE-D-23-05654 Review Comments VLS in Ukraine.docx [file pone.0290661.s002.docx]

**General**

This is a well written paper. It presents on an important issue in achieving the 90-90-90 – viral load suppression. By presenting prevalence of detectable viraemia and its correlates, the information could help drive efforts at achieving the 3^rd^ 95 of the UN strategy among HIV-positive PWIDs in Ukraine.

The comments I provide here are to help the reader of the paper when published to better understand the information provided

**Abstract**

Line 33/34. In describing the relationship between HIV-negative PWID and HIV-positive PWID, consider using the phrase “compared with” in place of the word “unlike”.

**Materials and methods**

- General: This section has all the information needed, but the arrangement can be confusing. It appears the methods described here are reported in a previous study by Sazanova et al (2020; <https://journals.plos.org/plosone/article?id=10.1371/journal.pone.0244572#pone-0244572-t002>). If this is the case, authors are advised to refer to the paper by Sazanova for details of the methods and endeavor to summarize it here, following the chronology and clarity in the referenced paper.
- However, I provide further specific comments below:

**Study setting and design**

- The chronological presentation of the paragraphs can be improved for better logical flow.
- The section includes sample size determination and calculation, which is not covered in the sub-heading. Consider a new section on sample size or expand the present sub-heading to include it – e.g “Study setting, design and sample”.
- Lines 71/73 – Part of the sentence here is a repetition of the first sentence in this section. Consider reviewing this sentence to remove redundancy.
- Lines 79/80 – Unless the aim here is to demonstrate the role of incentives in the recruitment, I would suggest that this issue is presented under the “ethical considerations” section.

**Data collection**

- General – one can deduce that what is presented here is a set of procedures used to collect data for the study. In chronology as presented, this includes blood collection and biomarker testing, and questionnaire-based interview. For better clarity, the authors may wish to consider presenting these separate paragraphs. Issues about how the data from the separate parts of the study was linked and referral services provided for HIV and HCV-positive participants can be discussed subsequently. But it is also important to mention what happened to HIV and HCV-negative participants. A sentence on pre and posttest counselling can be added in chronological flow with referral services to make the appropriate point.
- Line 82/83 – In the light of the general observation above, the authors may wish to revise the opening sentence to be more specific about the data collection algorithm alone. For example – “Data collection was achieved through three different mechanisms – **questionnaire-based interview,** **blood collection**, and **biomarker testing** for HIV, HCV and VL.” A chronology that presents the process followed in the field to a greater extent would provide more clarity.
- Line 83 – did the authors mean to say, “obtaining informed consent”? It feels like informed consent written alone does not say what was done.

**Ethical considerations**

Line 121 – I think the authors are missing the word “be” in the following phrase*: “procedures and was determined to research”*

Results

Line 215 – In presenting results on wage earning, it is important to be clear when mention is made of low or high wage. The tables (1 and 2) are clear of the income/wage classifications. Thus, if by “low monthly income” on line 215, the authors meant to say, “monthly income below minimum wage”, then using the right descriptor would help the reader more. This issue is observed in other parts of the paper for the authors’ consideration.

Lines 261/262 – It is not clear, the message that these phrases sought to convey – *“had average monthly income in reference to lower income level”* and “*had any educational level than completed school”*. The authors should kindly review and provide better clarity as necessary.

Discussion

Line 286 – While it is accurate that more than 11.1% of all PWIDs were reported to have DV in this paper, when compared to the actual reported estimate of 11.4%, this statement could lead the audience to wrong conclusions. Authors should consider revising this statement to avoid such risks.

Lines 316-319 – The two sentences here can be improved upon (Nearly 73% of HIV-positive PWID with DV generally did not engage in ART. This proportion among the young age group (<35 years old), subgroup with low education level, and those who reported about monthly income higher than on average ranged from 81%-85%.). Consider instead the following:

“Nearly 73% of HIV-positive PWID with DV generally did not engage in ART. Of this group, 81%-85% on average is characterized by young persons (<35 years old), those with low education, and those with reported higher than national average monthly income”

This is how I am reading the two sentences. The authors should consider the most appropriate revision though.

Lines 369/370 – This phrase in the last sentence can be revised for better clarity “*treatment service to sensitize their approaches to access populations with greater needs.”*
