# Supplementary material for: Population-level prevalence of detectable HIV viremia in people who inject drugs (PWID) in Ukraine: Implications for HIV treatment and case finding interventions
Source: PLoS One. 2023 Oct 26;18(10):e0290661. doi: 10.1371/journal.pone.0290661 (PMC10602286; doi:10.1371/journal.pone.0290661)
Supplement: Attachment — Submitted filename: Response_to_Reviewers.docx [file pone.0290661.s003.docx]

**Dear editors and reviewers!**

On behalf of all co-authors, I would like to extend our sincere appreciation for your time, effort, and expertise in reviewing our manuscript titled, *“Population-level prevalence of detectable HIV viremia in people who inject drugs in Ukraine: implications for HIV treatment and case finding interventions”*. Your valuable comments and constructive feedback have helped us greatly in improving the quality of the manuscript. We are extremely grateful for your contribution to this project. Detailed responses to your comments provided in the table below.

| **Editors’ comments:** | **Co-authors responses:** |
| --- | --- |
| Please ensure that your manuscript meets PLOS ONE's style requirements, including those for file naming. The PLOS ONE style templates can be found at  https://journals.plos.org/plosone/s/file?id=wjVg/PLOSOne_formatting_sample_main_body.pdf and  <https://journals.plos.org/plosone/s/file?id=ba62/PLOSOne_formatting_sample_title_authors_affiliations.pdf> | Thank you! We checked and updated the manuscript accordingly. |
| 2. Thank you for stating the following financial disclosure:  “The bio-behavioral surveillance study has been supported by the President's Emergency Plan for AIDS Relief (PEPFAR) through the Centers for Disease Control and Prevention (CDC) under the terms of Cooperative Agreement NU2GGH000840 implemented by Alliance for Public Health.”  At this time, please address the following queries:  a)        Please clarify the sources of funding (financial or material support) for your study. List the grants or organizations that supported your study, including funding received from your institution.  b)        State what role the funders took in the study. If the funders had no role in your study, please state: “The funders had no role in study design, data collection and analysis, decision to publish, or preparation of the manuscript.”  c)        If any authors received a salary from any of your funders, please state which authors and which funders.  d)        If you did not receive any funding for this study, please state: “The authors received no specific funding for this work.”  Please include your amended statements within your cover letter; we will change the online submission form on your behalf. | Financial disclosure was updated to include option “d” and was added to the cover letter. The financial disclosure is following:  *“The BBS has been supported by the President's Emergency Plan for AIDS Relief (PEPFAR) through the Centers for Disease Control and Prevention (CDC) under the terms of Cooperative Agreement NU2GGH000840 implemented by the Alliance for Public Health. The authors received no specific funding for this work.”*  Additionally, per your comment, we have deleted the financial disclosure from the manuscript text. |
| We note that you have indicated that data from this study are available upon request. PLOS only allows data to be available upon request if there are legal or ethical restrictions on sharing data publicly. For more information on unacceptable data access restrictions, please see http://journals.plos.org/plosone/s/data-availability#loc-unacceptable-data-access-restrictions | Thank you for the guidance. We included the dataset as the supplemental material. Please be informed that the dataset was restricted to the number of variables and cases that was used in analysis. |

| **Reviewers’ comments #1:** | **Co-authors responses:** |
| --- | --- |
| **General**  This is a well written paper. It presents on an important issue in achieving the 90-90-90 – viral load suppression. By presenting prevalence of detectable viraemia and its correlates, the information could help drive efforts at achieving the 3^rd^ 95 of the UN strategy among HIV-positive PWIDs in Ukraine.  The comments I provide here are to help the reader of the paper when published to better understand the information provided. | Thank you so much for your comments and suggestions. We tried to address all of them in the updated version of the manuscript. |
| **Abstract**  Line 33/34. In describing the relationship between HIV-negative PWID and HIV-positive PWID, consider using the phrase “compared with” in place of the word “unlike”. | Thank you for suggestion, it is corrected (Line 34 in track-changed version) |
| **Materials and methods**  General: This section has all the information needed, but the arrangement can be confusing. It appears the methods described here are reported in a previous study by Sazanova et al (2020; <https://journals.plos.org/plosone/article?id=10.1371/journal.pone.0244572#pone-0244572-t002>). If this is the case, authors are advised to refer to the paper by Sazanova for details of the methods and endeavor to summarize it here, following the chronology and clarity in the referenced paper. | The chronology of presented methods was updated according to reviewer’s suggestion and reference on previous paper with more detailed description of methods was provided (Line 74, Lines 91-137 in track-changed version) |
| **Study setting and design**   - The chronological presentation of the paragraphs can be improved for better logical flow. | The chronological presentation of the paragraphs was improved (Lines 91-137 in track-changed version) |
| - The section includes sample size determination and calculation, which is not covered in the sub-heading. Consider a new section on sample size or expand the present sub-heading to include it – e.g “Study setting, design and sample”. | The sub-heading was changed to “Study setting, design and sample” (Line 75 in track-changed version) |
| - Lines 71/73 – Part of the sentence here is a repetition of the first sentence in this section. Consider reviewing this sentence to remove redundancy. | The sentence was updated “We used the data from the BBS study among PWID, conducted in 30 cities in Ukraine from October 3rd to December 28th, 2017. The study collected data on behavioral practices with on-site testing for HIV and HCV infections and laboratory testing for HIV recency and VL.” Lines 76-79 in trach-changed version. |
| - Lines 79/80 – Unless the aim here is to demonstrate the role of incentives in the recruitment, I would suggest that this issue is presented under the “ethical considerations” section. | It was moved to Ethical Considerations sub-section, lines 147-149 in track-changed version. |
| **Data collection**   - General – one can deduce that what is presented here is a set of procedures used to collect data for the study. In chronology as presented, this includes blood collection and biomarker testing, and questionnaire-based interview. For better clarity, the authors may wish to consider presenting these separate paragraphs. - Issues about how the data from the separate parts of the study was linked and referral services provided for HIV and HCV-positive participants can be discussed subsequently. - But it is also important to mention what happened to HIV and HCV-negative participants. - A sentence on pre and posttest counselling can be added in chronological flow with referral services to make the appropriate point. | - The chronology was changed to represent study algorithm: questionnaire, rapid testing, lab testing. Each of them is presented as separate paragraphs Lines 90-122 in track-changed version. - The information was added “The HIV and HCV rapid testing results and laboratory VL testing results were merged with the data from the survey questionnaire using the study participants' unique codes.” Lines 123-124 in track-changed version. - The information was added “Additionally, all participants, including HIV-negative, received information about community-based HIV services for PWID available in the city and were referred there.” Lines 128-130 in track-changed version. - Information was added “All participants received pre-and post-test counseling by medical workers according to the national guidelines.” Lines 112-113 in track changed version. |
| - Line 82/83 – In the light of the general observation above, the authors may wish to revise the opening sentence to be more specific about the data collection algorithm alone. For example – “Data collection was achieved through three different mechanisms – **questionnaire-based interview,** **blood collection**, and **biomarker testing** for HIV, HCV and VL.” A chronology that presents the process followed in the field to a greater extent would provide more clarity. | Opening sentence was added “Data collection was achieved through three different mechanisms: questionnaire-based interview, biomarker testing for HIV, hepatitis C (HCV), and laboratory testing of dry blood spots (DBS) for HIV recency and VL.” Lines 92-96 in track changed version. |
| - Line 83 – did the authors mean to say, “obtaining informed consent”? It feels like informed consent written alone does not say what was done. | The sentence was modified to get more clarity “The data collection teams obtained written informed consent from all participants before the study.” Lines 139-140 in track changed version. |
| **Ethical considerations**  Line 121 – I think the authors are missing the word “be” in the following phrase*: “procedures and was determined to research”* | Yes, it was a typo – corrected. Line 152-153 in track changed version. |
| Results  Line 215 – In presenting results on wage earning, it is important to be clear when mention is made of low or high wage. The tables (1 and 2) are clear of the income/wage classifications. Thus, if by “low monthly income” on line 215, the authors meant to say, “monthly income below minimum wage”, then using the right descriptor would help the reader more. This issue is observed in other parts of the paper for the authors’ consideration. | Categories were changed to have more clarity throughout the paper. Lines 247-248, 249, 277-279, 295-298 in track changed version. |
| Lines 261/262 – It is not clear, the message that these phrases sought to convey – *“had average monthly income in reference to lower income level”* and “*had any educational level than completed school”*. The authors should kindly review and provide better clarity as necessary. | Categories were changed to have more clarity throughout the paper. Lines 247-248, 249, 277-279, 295-298 in track changed version. |
| Discussion  Line 286 – While it is accurate that more than 11.1% of all PWIDs were reported to have DV in this paper, when compared to the actual reported estimate of 11.4%, this statement could lead the audience to wrong conclusions. Authors should consider revising this statement to avoid such risks. | 11.1% was a typo. The correct result is 11.4% which is calculated on weighted data. Corrected. |
| Lines 316-319 – The two sentences here can be improved upon (Nearly 73% of HIV-positive PWID with DV generally did not engage in ART. This proportion among the young age group (<35 years old), subgroup with low education level, and those who reported about monthly income higher than on average ranged from 81%-85%.). Consider instead the following | Thank you for suggestion. The text was changed to “Nearly 73% of HIV-positive PWID with DV generally did not engage in ART. Of this group, 81%-85% on average is characterized by young persons (<35 years old), those with low education, and those with reported higher than national average monthly income.”. Lines 353-356 in track changed version. |
| “Nearly 73% of HIV-positive PWID with DV generally did not engage in ART. Of this group, 81%-85% on average is characterized by young persons (<35 years old), those with low education, and those with reported higher than national average monthly income”  This is how I am reading the two sentences. The authors should consider the most appropriate revision though. | Thank you for suggestion. The text was updated accordingly Lines 353-356 in track changed version. |
| Lines 369/370 – This phrase in the last sentence can be revised for better clarity “*treatment service to sensitize their approaches to access populations with greater needs.”* | Text was updated “The study identified key predictors of DV that might inform HIV prevention, case finding, and treatment service and help to sensitize program approaches to access HIV-positive PWID subgroups that experience a larger risk of DV”. Lines 409-411 in track changes version. |
|  |  |
| **Reviewers’ comments #2:** | **Co-authors responses:** |
| Line 46 – Add a sentence on the total number of PLHIV who are on ART | The information was added in percentages.  “Globally, the estimated size of people living with HIV (PLHIV) reached 38.4 million in 2021. Of all PLHIV, 75% (66-85%) were accessing ART, and 68% (60-78%) were virally suppressed.”  Lines 47-49 in track changes version. |
| Line 52 – Give an estimate of the total number of PWIDs in Ukraine before you write the proportion of these PWIDs that have HIV | The estimate of the PWID size was added:  “According to the latest estimates, there were 350,300 PWID in Ukraine, and 22.6% had HIV.”  Lines 54-55 in track changes version. |
| Data collection – State the protocol used for the data collection for PWIDs who already knew their HIV status and were already on ART. Did you repeat HIV rapid tests for them? | Yes, the full study algorithm was used for all participants despite their previous knowledge pf HIV or ART (Lines 103-104 in tack changed version). Data collection team has no access to verify self-reported information in the medical databases. |
| Results – For clients with detectable viral loads, compare if duration of ART treatment has a significant relationship with the level of viremia. | Thank you, we checked this relationship using categorical variable of time on ART (<6 months and => 6 months). Only categorical variable was available for analysis. |

Thank you again for your valuable input.

*Sincerely,*

*Yana Sazonova*
